# Template-Assisted Synthesis of Luminescent Carbon Nanofibers from Beverage-Related Precursors by Microwave Heating

**DOI:** 10.3390/molecules24081455

**Published:** 2019-04-12

**Authors:** Clara Deeney, Eoin P. McKiernan, Samir A. Belhout, Brian J. Rodriguez, Gareth Redmond, Susan J. Quinn

**Affiliations:** 1 School of Chemistry, University College Dublin, Dublin 4 N2E5, Ireland; clara.deeney@ucdconnect.ie (C.D.); eoin.mc-kiernan@ucdconnect.ie (E.P.M.); Samir.belhout@ucd.ie (S.A.B.); 2 School of Physics, University College Dublin, Dublin 4 N2E5, Ireland; brian.rodriguez@ucd.ie

**Keywords:** carbon fiber, luminescent, carbon nanofiber, beverage, templated synthesis, microwave

## Abstract

Luminescent carbon nanomaterials are important materials for sensing, imaging, and display technologies. This work describes the use of microwave heating for the template-assisted preparation of luminescent carbon nanofibers (CNFs) from the reaction of a range of beverage-related precursors with the nitrogen-rich polyethyleneimine. Highly luminescent robust carbon fibers that were 10 to 30 μm in length and had a diameter of 200 nm were obtained under moderate conditions of temperature (250–260 °C) and a short reaction time (6 min). The high aspect ratio fibers showed wavelength-dependent emission that can be readily imaged using epifluorescence. The development of these multi-emissive one-dimensional (1D) carbon nanomaterials offers potential for a range of applications.

## 1. Introduction

In the past 15 years, microwave irradiation has emerged as a powerful tool for the synthesis and functionalization of carbon nanomaterials [1]. This has included the microwave synthesis of carbon nanotubes [2,3], graphitic hollow carbon nanospheres [4], and the exfoliation of graphite to yield graphene [5]. Microwave irradiation has also been used to introduce functional groups at the surface of carbon nanotubes [6], nanohorns [7], and graphene oxide [8]. The key advantage of microwave heating compared to other methods is the access to rapid contactless direct heating that can be switched on or off instantaneously. The use of microwave methods in this way has provided alternatives to harsh reagents, such as acids, allowed the selective heating of reagents, and greatly reduced the reaction times [1].

Luminescent carbon nanomaterials have been widely investigated for their applications in imaging, displays, and sensing. Luminescent carbon dots (CDots), which typically have sizes below 10 nm, have attracted significant interest in recent years due to their biocompatibility and tunable optical properties, with emission observed across the visible spectrum [9,10]. These nanoparticles are routinely prepared via the carbonization of a wide range of small molecule precursors, including bio-organic acids, sugars, alcohols, and foodstuffs [11,12,13,14,15,16,17]. Furthermore, CDots have also been extracted from commercially available foods and beverages, including pilsner beer [18], Nescafé Original instant coffee [19], and baked goods [13]. Generally, the methods used to prepare CDots include laser ablation, solvothermal synthesis, electrochemical methods, and heating under reflux [20,21,22,23]. However, microwave synthesis has proven extremely effective for CDot synthesis with over 900 reports in the literature since 2003. The widespread application of microwave heating for CDots is due to the ready access to carbonization under conditions of rapid heating and increased pressure [15,24,25].

**Scheme 1:**
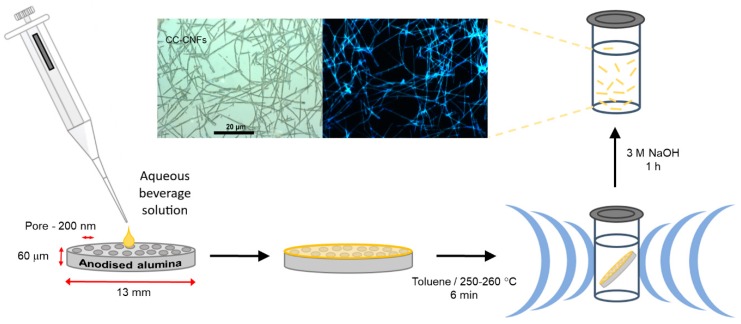
Overview of the synthesis of luminescent carbon nanofibers via the microwave-assisted hydrothermal pyrolysis of beverage precursors.

Microwave heating has also been used to prepare carbon fibers [26]. These materials are of interest due to their potential for energy storage as coatings and as catalyst supports [27,28,29]. The ability to prepare light-activated and surface-responsive carbon fibers is expected to expand this capability through potential application as environmental sensors and improved coatings. We recently reported the first synthesis of luminescent carbon nanofibers and demonstrated their application for the sensing of metal ions [30]. This was achieved by employing a templated microwave synthesis to perform the reaction of citric acid and polyethyleneimine (PEI); the microwave synthesis of CDots using these precursors had previously been reported. By translating this reaction to a membrane template, rapid microwave heating was found to result in the formation of blue-emitting nanofibers, which could be isolated in solution following the base digestion of the membrane template, as shown in Scheme 1. Shortly after this, improved carbon nanotube growth inside an anodic aluminum oxide template using microwave radiation was also reported [31], which further demonstrates the potential of combining templated synthesis and microwave heating. The mechanism of the formation of the luminescent carbon fibers involved the microwave-assisted hydrothermal carbonization of the precursors. The structure of the template will influence the overall dimensions of the materials, while the material features are expected to be influenced by the temperature and the nature of the molecular precursors. The choice of precursor has been found to influence the optical properties of luminescent CDots with nitrogen containing molecules including urea, ethylene diamine, and polyethyleneimine, resulting in improved luminescence [16,32]. In this report, we now demonstrate the general applicability of the template-assisted microwave method to the preparation of luminescent nanofibers from the reaction of a range of beverage-related precursors with PEI, and describe their luminescent optical properties.

## 2. Results and Discussion

Inspired by the literature on CDots, a range of food/beverage-related precursors were chosen to be investigated. These included malic acid (a constituent of apples), which has recently been used to prepare CDots for super-resolution imaging applications [33], and the related lactic acid (milk) for comparison. Four beverages were also considered, including lemon juice, orange juice, and Coca-Cola, all of which have been used to prepare CDots [12,34,35]. In addition, grapefruit juice was chosen, which has not yet been reported in the synthesis of CDots. The structures of some of the key molecular components of these beverages together with malic and lactic acid are shown in Scheme 1.

### 2.1. Carbon Nanofibers Prepared from Malic Acid (MA-CNF)

**Figure 1:**
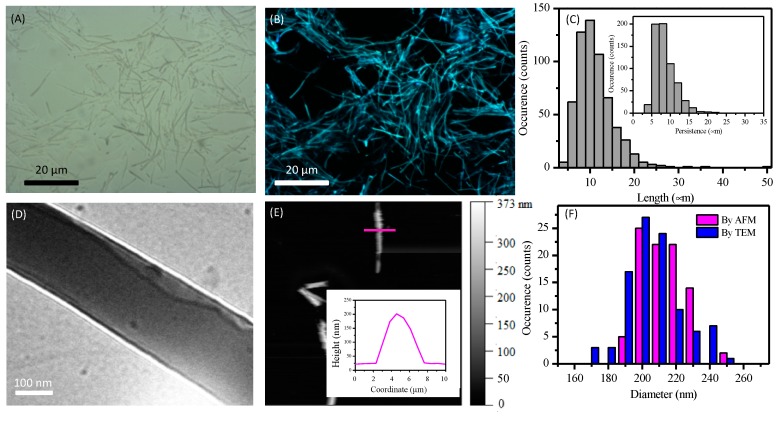
(**A**) Bright-field image of malic acid carbon nanofibers (MA-CNFs) under 100× magnification and (**B**) corresponding epifluorescence (λ_ex._ 365–420 nm λ_col_ > 430 nm) image. Drop cast from aqueous suspension onto glass. (**C**) Length distribution (and persistence length distribution—inset) of MA-CNFs derived from a range of epifluorescence images. (**D**) TEM image of a MA-CNF on a holey carbon substrate. (**E**) Atomic force microscopy (AFM) height trace of MA-CNFs in AC mode on glass and inset showing exemplar line trace. (**F**) Diameter distribution of MA-CNFs by TEM and AFM.

The template-assisted method that was previously developed for the preparation of luminescent citric acid CNFs [30] was first investigated for an aqueous solution of malic acid in the presence of PEI (800 MW). The precursor solution was deposited onto an anodized alumina filter membrane with a 200-nm nominal pore diameter and 60-μm length, and allowed to load by gravity over approximately 30 min. The membrane was then transferred to a glass reaction vial containing 2 mL of toluene, which was sealed and subjected to microwave heating at 250 °C under 16 bar pressure for 6 min. After the reaction, the color of the membrane was found to have changed from a white to a brownish color, as shown in Figure S1. The reaction of the MA was found to require a higher temperature than that used to previously prepare CNFs from citric acid and PEI (200 °C). Removal of the template using 3 M of aqueous NaOH and washing by dialysis against water gave a percentage yield of 8.4 ± 2.4%. The yield was calculated based on the dimensions of the membrane template and assuming a CNF density of amorphous carbon of 2.0 g/cm^3^, which has been used for the solvent-based synthesis of CDots and CNFs under similar temperature conditions [36,37]. Bright-field images under 100× magnification revealed the successful formation of carbon nanofibers (MA-CNFs) as shown in Figure 1A, which were found to emit blue luminescence when excited between 365–420 nm, as shown in Figure 1B. Analysis of the brightfield images using ImageJ software revealed the average nanofiber length to be 10 ± 4 µm, (*n* = 600), with a persistence length of 8 ± 3 µm, (*n* = 650), as shown in Figure 1C. The difference in the value of the average length and that of the average membrane pore length of 60 μm may be due to incomplete filling of the pores due to poor wetting or some breakage during the membrane digestion process and subsequent workup. However, the isolated fibers were found to be stable to continuous bath sonication for 1 h, with no apparent increase in fragmentation or damage detected under 100× magnification, as shown in Figure S2. Interestingly, when the synthesis of MA-CNFs was repeated under solvothermal conditions in a bomb reactor at 250 °C for 12 h, the fibers were found to be less well defined, with reduced brightness relative to those prepared under microwave irradiation, as shown in Figure S3. This suggests that the rapid heating conditions that occur in microwave synthesis are important for the structural integrity and emission intensity of the luminescent nanofibers.

Transmission electron microscopy (TEM) images showed the MA-CNFs edges to be clean with no decoration with smaller particles or frayed edges, as shown in Figure 1D and Figure S4. This suggests that the emission seen in the epifluorescence images is intrinsic to the MA-CNF, and is not a result of aggregated CDots or surface decoration by CDots. The TEM images also show regions of hollows apparently distributed randomly through some MA-CNFs. This may be due to the presence of air bubbles in the solution during the synthesis. The presence of these hollows suggests that the preparation of porous luminescent materials may be possible by changing the wetting conditions. The diameter distribution of the cross-section measured by transmission electron microscopy (TEM) taken at 250-nm increments was found to be quite uniform with an average of 201.1 ± 27 nm (*n* = 100), as shown in Figure 1F. Height analysis of the atomic force microscopy (AFM) images yielded an average diameter of 217 ± 32 nm (*n* = 90), which is in close agreement with that obtained from the TEM, as shown in Figure 1E,F. These values are also in good agreement with the average diameter of the membrane pores, which was determined by scanning electron microscopy (SEM) analysis of the empty anodized alumina membrane, which was measured as 213 ± 31 nm (*n* = 1087), as shown in Figure S5. A summary of the dimensions of the MA-CNF determined using different methods is given in Table S1.

**Figure 2:**
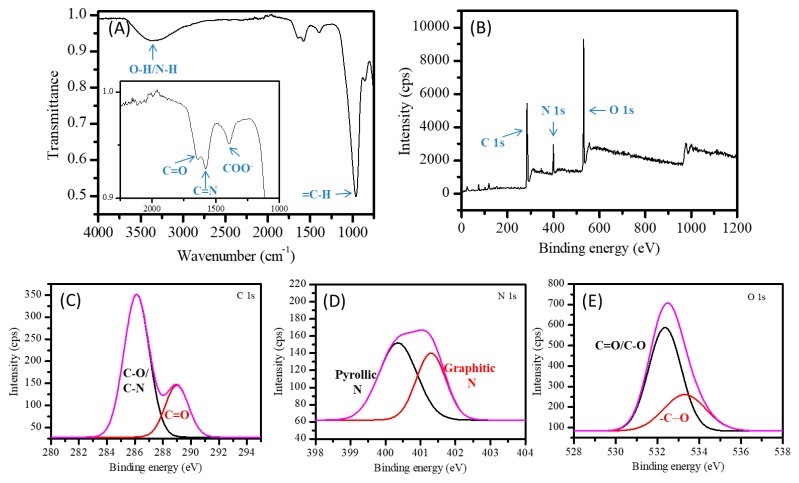
(**A**) Solid state Fourier transform infrared (FTIR) spectrum of MA-CNFs dried under reduced pressure. (**B**) X-ray photoelectron spectroscopy (XPS) full-range spectrum of MA-CNFs drop cast sequentially from aqueous suspension. (**C**–**E**) High-resolution XPS spectra of C 1s, N 1s, and O 1s regions, respectively.

Infrared characterization of the MA-CNFs showed stretching bands for N−H at 3361 cm^−1^ and the acid O−H at 2850 cm^−1^ with bands due to C=O, COO–, and the imine C=N stretch observed at 1670 cm^−1^, 1390 cm^−1^, and 1640 cm^−1^ respectively; see Figure 2A. X-ray photoelectron spectroscopy (XPS) was used to determine the chemical composition of the MA-CNFs, which was found to be 62.0% carbon, 11.0% nitrogen, and 27% oxygen. The high-resolution analysis of the XPS data was used to provide information on surface functionality. Three peaks were recorded at 286 eV, 401 eV, and 532 eV corresponding to the C 1s peak, N 1s peak, and O 1s peak, respectively (Figure 2 B), which are similar to those previously recorded for CDots prepared from malic acid [33,38]. The C 1s peak is composed of contributions from C−O/C–C at 286.1 eV and C=O at 288.9 eV; see Figure 2C. In contrast to the XPS spectra previously reported for the fibers prepared using citric acid, the presence of C–C/C=C was not observed in the XPS spectrum, although it is expected to contribute weakly to the envelope. A similar reduction in the C=C contribution has been observed for the XPS spectra recorded for Cdots prepared from malic acid as compared to citric acid; see Figure 2C [38]. The N 1s peak indicates the presence of pyrrolic (400.3 eV) and graphitic N (401.3 eV), while the O 1s peaks at 532.4 eV and 533.2 eV are assigned to C=O and ester-like –O–C groups; see Figure 2D,E [39,40,41]. The slightly higher value in the C=O band assignment here compared to that reported for Cdots prepared from carbohydrates [39] is likely due to the a contribution from C–O species as previously observed [41] The Raman spectrum recorded for the fibers shows no defined D or G band signal, and combined with no apparent lattice structure in TEM, it suggests that the carbon composition of the MA-CNF is amorphous in nature; see Figure S6 [42].

**Figure 3:**
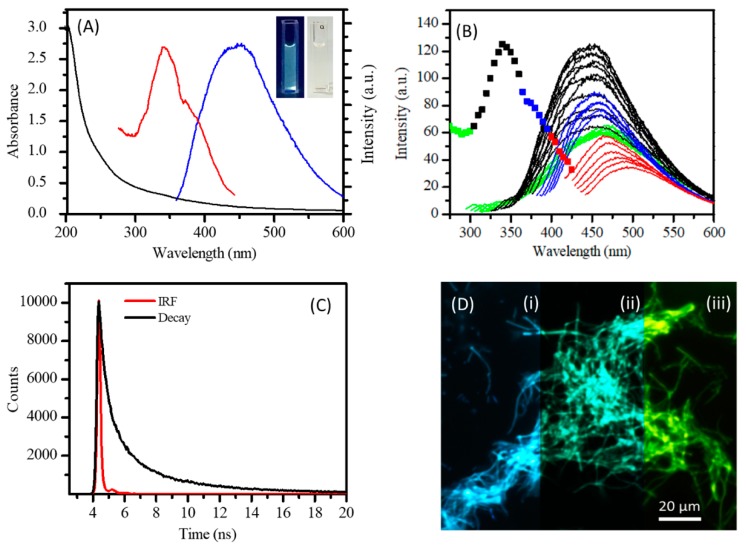
(**A**) Absorbance (black) emission at λ_ex_ 345 nm (blue) and excitation at λ_em_ 455 nm spectra of MA-CNFs in aqueous suspension. Inset shows suspension under 365-nm illumination and ambient lighting. (**B**) Excitation wavelength-dependent emission spectra of MA-CNFs at 5-nm increments from λ_ex_ 274 nm to 425 nm (λ_ex_: 275–300 nm green, 305–360 nm black, 365–390 nm blue, and 395–425 nm **red**) and the associated extrapolated excitation spectrum in squares. (**C**) Fluorescence lifetime decay of MA-CNFs in aqueous suspension λ_ex_ 365 nm λ_col_ 455 nm. (**D**) Epifluorescent images captured under (**i**) λ_ex._365–420 nm, λ_col_ > 430 nm; (**ii**) λ_ex._390–407 nm, λ_col_ > 407 nm; and (**iii**) λ_ex._450–490 nm, λ_col_ > 490 nm.

The MA-CNFs were found to have an unstructured absorption across the visible region of the spectrum, as shown in Figure 3A. Excitation of the sample at 345 nm resulted in a broad emission (full-width half-maximum, or FWHM = 145 nm) centered at 455 nm, which is greater than that previously obtained for citric acid CNFs; see Figure S7 [30]. The excitation spectrum showed a maximum of 340 nm, which represents a significant Stokes shift of 115 nm. The emission of the MA-CNF was found to be highly dependent on the excitation wavelength with the emission at 500 nm detected upon visible light excitation; see Figure 3B. This observation of excitation wavelength-dependent emission is in contrast to the largely excitation-independent emission seen in CA-CNFs, which is characteristic of molecular based emission [43,44]. This suggests that there may be multiple molecular-like species contributing to the emission [45,46]. The quantum yield of MA-CNFs was found to be 2.7% by integration sphere, which is notably lower than that of CA-CNFs (4.8%) and lower than CDots made from bio-organic acid and amine precursors [16]. The data obtained from fluorescence lifetime measurements fitted well to a biexponential decay with a short-lived species of 1.4 ns and a contribution from a longer-lived component at 7.7 ns. The broad (blue to yellow) wavelength-dependent emission was readily captured by recording the epifluorescence images under different conditions of excitation and collection; see Figure 3D and Figure S8.

The surface state nature of CDot emission is typically sensitive to the surrounding environment and has been exploited for numerous sensing applications [47,48,49]. The MA-CNF emission was found to be sensitive to pH with changes in both intensity of emission (up to 60%), and the position of the λ_max_ (up to 20-nm redshift from 455 nm) observed; see Figure S9A [50,51]. The emission response was found to be reversible upon cyclization from pH 7 to pH 1 to pH 13, which suggests the role of protonation and deprotonation of surface groups; see Figure S9B. The treatment of MA-CNFs with sodium borohydride caused a shift in the wavelength of maximum emission from 455 nm to 425 nm with an increase in the intensity of 155 ± 15%; see Figure S10. Similar changes have been observed upon CDot reduction and were attributed to the reduction of C=O and C=N functional groups [46]. The results from these two studies serve to demonstrate the ability to modulate the emission of these CNFs. The method to prepare MA-CNFs was also successfully used to prepare luminescent CNFs by reacting lactic acid and PEI under identical conditions of microwave synthesis and purification; see Tables S3 and S4. These nanofibers exhibited very similar properties to the MA-CNFs; see Figure S11.

### 2.2. Carbon Nanofibers Prepared from Commercially Available Beverages

**Figure 4:**
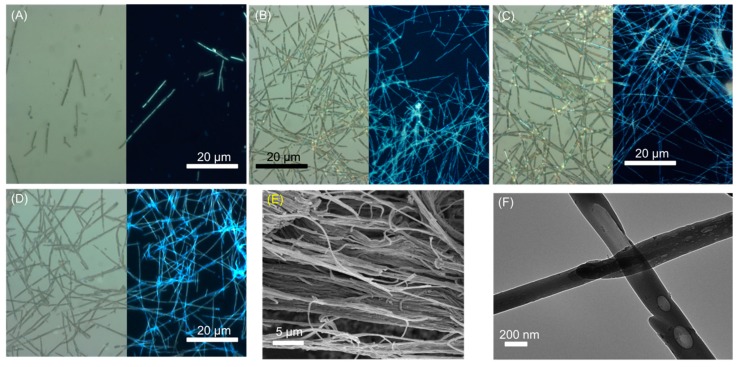
(**A**–**D**) 100× mag images of Lj-CNFs, Oj-CNFs, Gj-CNFs and CC-CNFs respectively under conditions of bright-field and epiflourescence illumination (λ_ex_ 365–420 nm, λ_col_ > 430 nm). Scale applies to all. (**E**) SEM image of gold-sputtered CC-CNFs. (**F**) TEM image of CC-CNFs drop cast from aqueous suspension onto a lacey carbon substrate and dried in air. Lj: lemon juice, Oj: orange juice, Gj: grapefruit juice, CC: Coca-Cola.

Having prepared CNFs from the molecular constituents of citrus fruit (citric acid) [30], apple (malic acid), and milk (lactic acid), we next considered whether the use of available foodstuffs could be used. This study investigated the use of orange juice (Oj), grapefruit juice (Gj), lemon juice (Lj), and the soft drink Coca-Cola (CC). The relationship between the chemical structure and luminescence properties of Cdots is not yet fully resolved [16,20], with the origin of the observed luminescence expected to be derived from a combination of surface molecular-based species and the core carbon state [16]. In particular, the influence of the population of amines at the surface has been observed to increase the wavelength of emission from blue to green [17]. The use of water-soluble precursors with hydrophilic groups is expected to influence the nature of the surface of the carbon fibers, which may be tuned by varying the temperature and ratio of amine-containing groups. As such, the luminescence properties of the microwave-assisted hydrothermal carbonization of readily available beverage precursors is expected to be influenced by: (a) the major constituents of the beverages, including sucrose, glucose, fructose, citric acid, and ascorbic acid, as well as the more conjugated furanocoumarin and caffeine, and (b) the presence and absence of PEI. To do this, a 10X juice/soft drink concentrate (by evaporation) was reacted (i) with and (ii) without PEI at 260 °C for 6 min; see Tables S3 and S4. In all the cases, the reaction conditions resulted in a darkening of the membrane appearance, which was taken as an indication of the reaction proceeding. The bright-field images of Lj-CNF, Gj-CNF, Oj-CNF, and CC-CNF prepared with PEI revealed highly persistent and relatively long CNFs that showed little fragmentation; see Figure 4A–D. The epifluorescence images showed strong emission for the drinks-based CNFs prepared using PEI; see Figure 4A–D. In contrast the CNFs prepared without PEI (CNF(no-PEI)) were only weakly emissive; see Figure S12.

The lengths of the drink-CNFs all show a relatively long total length (17–37 μm) compared to MA-CNFs (11 μm); see Table 1 and Figure S13. The longest CNFs measured were for Oj without PEI and Gj without PEI (32 ± 11 μm and 37 ± 10 μm, respectively). Each of the drink-CNFs had instances of nanofibers of 60 μm, which is the reported full length of the membrane template. This suggests that the reaction mixture is fully filling the template, but importantly, no CNFs longer than 60 μm were seen, which implies that the drink-CNFs are being formed inside the template pores and not crystalizing on the surface of the membrane. This is again illustrated by the SEM image of the gold-sputtered membrane after partial digestion for CC-CNF, which shows a dense forest of fibers; see Figure 4E. The TEM image of CC-CNFs show the surfaces to be smooth with no indication of any CDot coating; they also reveal the presence of structural hollows along the lengths of the CNFs, which have been observed for the MA-CNF, as shown in Figure 4F.

The chemical composition of the drink-CNFs was measured by combustion elemental analysis, which showed that when PEI was used, the N composition was approximately 13% and decreased to <2% for the CNFs prepared without PEI; see Table S5. These results were reflected in the Fourier transform infrared (FTIR) of the drink-CNFs, which showed a greater contribution to the amide stretch at 1633 cm^−1^ for samples containing PEI; see Table S6 and Figure S14.

**Figure 5:**
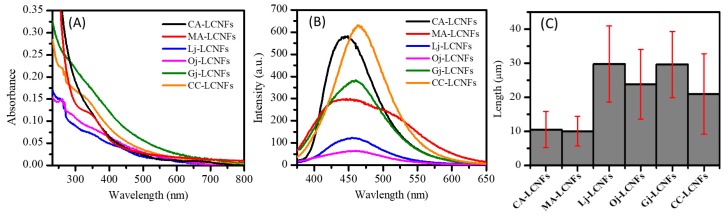
(**A**) Absorbance spectra of all the CNF samples in aqueous suspension. (**B**) Emission spectra of all the CNF samples in aqueous suspension λ_ex_ 350 nm, Abs 0.1 at 350 nm for all samples. (**C**) Length comparison of a CNF samples as determined by optical microscopy.

**Figure 6:**
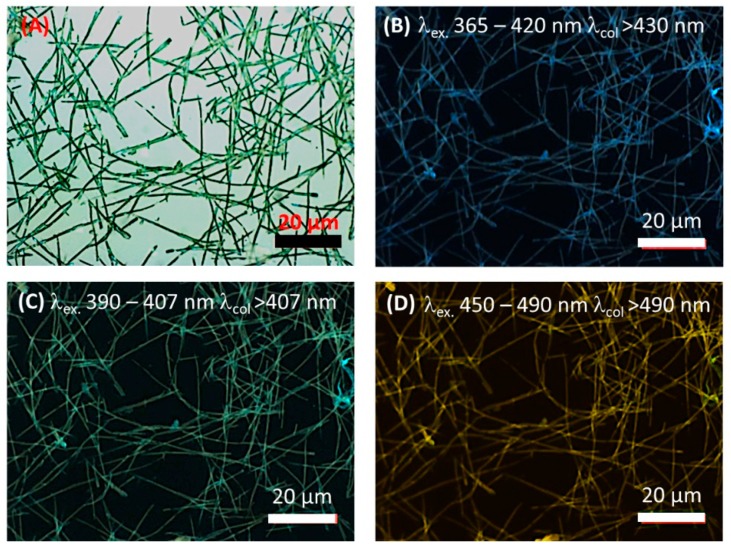
100× magnified images of CC-CNFs under (**A**) bright-field illumination and (**B**–**D**) epifluorescence illumination as shown.

The absorbance spectra of the CNFs prepared with PEI show some structured absorption below 400 nm with a gradual decrease of absorbance from 400 to 800 nm; see Figure 5A. In contrast, the spectra obtained for the reaction without PEI are largely featureless; see Figure S14. The epifluorescence images revealed the PEI samples to be more emissive, and these samples were found to display excitation-dependent emission; see Figure S16 and Table S7. The maximum emission was recorded at 350-nm excitation, which resulted in emission centered between 455–464 nm, with the greatest emission intensity found for CC-CNF. Notably, the FWHM of 50 nm of the excitation spectrum is narrower for all the PEI drink-CNFs than the MA-CNF (90 nm), see Figure 5B,C. In all the cases, excitation at 400 nm resulted in weaker green emission centered at ca. 500 nm. In line with the observations made by epifluorescent imaging, the drink-CNFs prepared without PEI were less emissive. Weak luminescence was observed for Oj-CNF(no-PEI) and Gj-CNF(no-PEI) samples, but the Lj-CNF(no-PEI) and CC-CNF(no-PEI) were non-luminescent; see Figure S17. This observation reflects previous reports that indicate the requirement of nitrogen-containing precursors to provide strong emission [11]. A comparative summary of the emission intensity and the fiber lengths of the different systems is given in Figure 6B,C, and highlights the improved structural and emissive properties the CC-CNF and Gj-CNF over the MA-CNF sample. As all three CNFs were prepared using PEI, it is reasonable to conclude that the added molecular components present in the fruit juice are important. Interestingly, sugar molecules such as fructose are present in high concentrations in fruit juices, and have recently been used to prepare CDots [49].

Finally, the wavelength-dependent emission demonstrated by the drink fibers was also observed under epifluorescence imaging and recorded under different conditions of filtered light excitation and collection, which show the dramatic range of emission from the CC-CNFs; see Figure 6.

## 3. Materials and Methods

Carbon fibers were prepared by dropping the precursor solution (40 μL) onto the surface of a 0.2-μm pore diameter Anodisc (anodized alumina) membrane and allowing it to wet the pores for approximately 30 min. The filled membrane was transferred to a 10-mL glass microwave vessel and submerged in 2 mL of toluene, which had not been degassed, and the vial was sealed with a silicone septum held in place by a PEEK-made cap. The sample was microwaved at 6 min (see the temperature and pressure conditions in Table S4). The membrane was removed from the toluene and left to dry in air. Any excess solid carbon was removed from the upper and lower surface of the membrane using a blade. Then, the membrane was placed in a 2-mL Eppendorf vial with 1 mL of 3M NaOH, and the vial was wrapped in aluminum foil to keep it in the dark while it shook for 1 h to dissolve the membrane. Sonication was used for 15 min to separate the nanofibers (not needed for MA-CNFs). Then, the suspension was centrifuged at 11,000 for 3 min, and the supernatant discarded and replaced with deionized water to remove the NaOH; this centrifuge step was repeated five times. In the case of MA-CNF, the suspension was dialyzed in a 3500 MWCO (molecular weight cut-off) membrane in a 2-L water bath for at least 1 h, with 10 water changes. All the samples were stored in aqueous suspension in the dark. The MA-CNF yield was calculated from the experimental weight of the nanofiber sample and the theoretical yield as determined by the number of pores recorded from the SEM image of the empty template (1.84 (±0.22) × 10^9^), Figure S5. The volume of the pore (1.9 (±0.23) × 10^−18^ m^3^) was valued based on the width determined using a diameter of 237.3 ± 39.2 nm and a height of 60 μm, as specified. The equivalent carbon mass of 8.7 mg was then calculated assuming the nanofibers have the density of amorphous carbon, 2.0 g cm^−3^.

### 3.1. Characterisation of Dispersed Carbon Fibers

The spectroscopic properties of the as prepared aqueous carbon nanofiber aqueous suspension was measured in a low volume (500 µL) quartz cuvette. Absorbance was measured on a Cary 60 and excitation and emission were measured on a Cary Eclipse. Emission spectra for suspensions of carbon fibers (absorbance at 350 nm at 0.1) were taken. These carbon fiber suspensions were spun down via centrifugation and resuspended in buffers of known pH (pH 1, pH 3, pH 5, pH 7, pH 9 and pH 11), and the emission spectra was repeated. For emission switching, the emission spectrum was taken a suspension of carbon fibers in water (pH7). Conc. HCl was added resulting in pH of one (emission spectrum was taken) followed by addition of conc. NaOH until the pH had reached 11 (emission spectrum was taken). Lifetime measurements were made on a Jobin Yvon Horiba FluoroCube-01-NL with excitation 365 nm, collection wavelength was 455 nm. The Instrument Response Frequency was found using Ludox colloidal silica in water. Quantum yield measurements. The as prepared aqueous carbon nanofiber aqueous suspension was measured in a 3 mL quartz cuvette. With absorbance 0.1 at excitation wavelength. The baseline was taken with deionized H_2_O and an integration sphere was used to collect the absorbance and emission counts.

### 3.2. Characterisation of Deposited Samples

AFM images were taken in amplitude modulation mode in air using an Asylum Research MFP-3D with a PPP-NCH tip (Nanosensors, Neuchatel, Switzerland). The instrument was Asylum Research MFP-3D AFM, at a scan rate of 1 Hz. Raman measurements was performed on a SENTERRA dispersive Raman microscope (Brunker optics) with a 785 nm laser. Settings applied were 1 mW power, 10 s collection times and 10 accumulations. The sample was deposited from aqueous suspension onto a cleaned CaF_2_ plate, a total of 6 mL as prepared nanofiber was dropped sequentially onto the same area in 10 µL aliquots. A nanofiber sample was dried under reduced pressure. The IR spectra were recorded for the dried using a Brunker Alpha-Platinum ATR, with a background acquired in air. X-ray Photon Spectroscopy (XPS) was performed using Kratos Axis Ultra XPS system, Kratos Analytical Ltd. (Manchester, UK) using a monochromatic Al K α1,2 source (150 W 15 kV and pass energy 10 eV for the high resolution scans and 160 eV for the wide scan). Spectra were analysed using Vision software (by Kratos). The peak positions were referenced to the C 1s hydrocarbon peak at 284.8 eV and a linear subtraction was applied. The peaks were fitted using an asymmetric GL(30) (Gaussian/Lorentzian product). The sample holder was sonicated in ethanol for 30 min and dried in air to clean. 10 μL of the carbon fiber suspension at a time were deposited into the middle of the sample holder and dried in air at 60 °C. This was repeated until a total of 3 mL was deposited and dried to ensure no gaps between the fibers, revealing the sample holder.

SEM measurements were taken using a FEI Quanta 3D FEG DualBeam (FEI Ltd., Hillsboro, OR, USA). Once the membrane was removed from the microwave and dried in air, the ‘top side’ was identified (more uniform pore distribution in anodizing process—appears shinier) and this side was scratched only. The underside was stuck to an adhesive carbon pad and approx. 100 μL 3 mol L-1 NaOH was dropped onto the membrane and left for 25 min. The membrane (still attached to the pad) was then rinsed with clean deionized H_2_O and left to dry. The carbon pad was then stuck to a sample holder. Samples were sputter coated with gold, using an Emitech K575X Sputter CoatingUnit, to prevent surface charging by the electron beam. Transmission Electron Microscope (TEM) and Scanning Transmission Electron Microscope (STEM) images were recorded on a FEI TITAN TEM and a JEOL2100 TEM at acceleration voltage 300 kV. The nanofibers were deposited from aqueous suspension onto a holey carbon substrate and dried in air under desiccation conditions.

## 4. Conclusions

Microwave heating has become an increasingly important method for the development of new carbon nanomaterials. Here, we have demonstrated the general applicability of microwave heating and template synthesis to prepare luminescent carbon fibers from a range of beverage-related precursors. Robust CNFs were obtained under conditions of moderate microwave heating and a short reaction time. The structural quality and emission properties were observed to be improved compared to fibers obtained using the templated under solvothermal synthesis conditions. The CNFs prepared from fruit juice and Coca-Cola showed significant improvements in persistence length and the emission properties over those prepared from malic and lactic acid, which may arise due to the presence of sugar molecules. Interestingly, the use of a nitrogen-rich polyimine proved critical to ensure good luminescent, and future work will consider the role of other nitrogen containing precursors such as urea. The CNFs prepared were found to display excitation-dependent emission spanning from blue to green. Future work will consider the ability to tune this emission to access red-emitting materials. TEM measurements revealed pores in the CNF structures. The ability to tune the density of these may allow the development of porous luminescent carbon materials for sensing, as the MA-CNFs luminescence was found to be sensitive to pH, and CA-CNFs have been shown to be capable of metal ion sensing [30]. Here, microwave heating treatment of the solid material may be investigated, as this has been previously used to generate porous material from carbon solid precursors [1]. Going forward, the carbon luminescent fibers reported here may be incorporated into sensing devices to report on environmental changes, such as changes in pH and composite materials to act as catalyst supports.

## Figures and Tables

**Table 1:** Average lengths of drink-CNFs as measured by bright-field microscopy.

Sample	Length (μm)	*N*	Sample	Length (μm)	*n*
Lj-CNF	30 ± 11	208	Lj-CNF (no PEI)	N/A *	
Oj-CNF	24 ± 10	200	Oj-CNF (no PEI)	32 ± 11	200
Gj-CNF	30 ± 10	200	Gj-CNF (no PEI)	37 ± 10	220
CC-CNF	21 ± 12	200	CC-CNF (no PEI)	19 ± 11	200

* Due to lack of definition, Lj-CNFs could not be accurately measured.

## Supplementary Materials

The following are available online. **Figure S1.** Comparison of the appearance of the MA-precursor solution filled anodized alumina template before and after microwave heating. **Figure S2.** Bright field ×100 magnification images of MA-CNFs and the corresponding epifluorescence images (λex. 365–420 nm λcol > 430 nm). Images of MA-CNFs following 1h bath sonication under conditions of C Bright field and D λex.365–420 nm, λcol > 430 nm. Drop cast from aqueous suspension and dried in air. **Figure S3.** ×100 mag. epifluorescence images (under λex. 365–420 nm λcol > 430 nm) of MA-CNFs prepared by solvothermal methods drop cast from aqueous suspension and dried in air. **Figure S4.** TEM images of MA-CNFs. drop cast from aqueous suspension and dried in air. (A) Deposited on lacey carbon substrate (B) and (C) Deposited on holey carbon substrate. **Figure S5.** A SEM images of empty anodized alumina templates as used in the synthesis of carbon fibers. B Size distribution of the diameter of the pore size determined from the SEM images, n = 1087. **Figure S6.** A FTIR spectrum of MA-CNFs deposited on CaF2 from aqueous suspension. B Raman spectrum of MA-CNF, deposited on CaF2 from aqueous suspension. **Figure S7** Comparison of the emission spectra of the MA-CNFs with the previously reported citric acid based CNFs. **Figure S8.** ×100 mag. images of MA-CNFs drop-cast from aqueous suspension and dried in air under illumination conditions of A. Bright field B. Epifluorescent images captured under λex.365–420 nm, λcol > 430 nm C. λex.390–407 nm, λcol > 407 nm D. λex.450–490 nm, λcol > 490 nm. **Figure S9.** Response of MA-CNFs to changes in their solvent conditions. A. pH sensitivity in an aqueous environment. B. pH switching in an aqueous environment. Samples had abs of 0.1 at 350 nm. **Figure S10.** Effect of reduction by sodium borohydride on the emission and excitation of MA-CNF. **Figure S11.** ×100 mag. images of LA-LCNFs under (A) Bright field illumination and (B) Epifluorescence illumination λex. 365–420 nm λcol > 430 nm. **Figure S12.** ×100 mag. bright field and epifluorescence images (under λex. 365–420 nm λcol > 430 nm) of CNFs prepared without the addition of PEI in the precursor solution (A)(B) Lj-CNFs (C)(D) Oj-CNFs (E)(F) Gj-CNFs and (G)(H) CC-CNFs. **Figure S13.** Length distributions of drink-CNFs with and without PEI as measured by bright field images. **Figure S14**. FTIR spectra of drink-CNF both with and without the inclusion of PEI in the precursor solution. **Figure S15.** Absorbance spectra of non-PEI containing drink-CNFs in aqueous suspension. **Figure S16.** Excitation wavelength dependent emission spectra of all drink-CNFs prepared with PEI in aqueous suspension. **Figure S17.** Excitation wavelength dependent emission spectra of all drink-CNFs prepared without PEI in aqueous suspension. **Table S1.** Comparison for MA-CNF dimensions with CA-CNF dimensions. **Table S2.** FTIR assignment of MA-CNFs. **Table S3**. Reaction mixture composition for preparation of carbon fibers. **Table S4.** Microwave conditions for each of the CNF reactions. **Table S5.** Elemental analysis of drink-CNFs in atomic percent. **Table S6.** Peak assignments for FTIR of drink-CNFs **Table S7.** Wavelength positions of emission maxima for drink-CNFs as a function of their wavelength of excitation.
