# Supplementary material for: Template-Assisted Synthesis of Luminescent Carbon Nanofibers from Beverage-Related Precursors by Microwave Heating
Source: Molecules. 2019 Apr 12;24(8):1455. doi: 10.3390/molecules24081455 (PMC6515550; doi:10.3390/molecules24081455)
Supplement: Supplementary file 1 [file molecules-24-01455-s001.pdf]

Supporting Information

Template-assisted synthesis of luminescent carbon nanofibers from beverage related precursors by microwave heating

Clara Deeney,[a] Eoin McKiernan,[a] Samir A. Belhout,[a] Brian J. Rodriguez,[b] Gareth Redmond*[a] and Susan J. Quinn*[a]

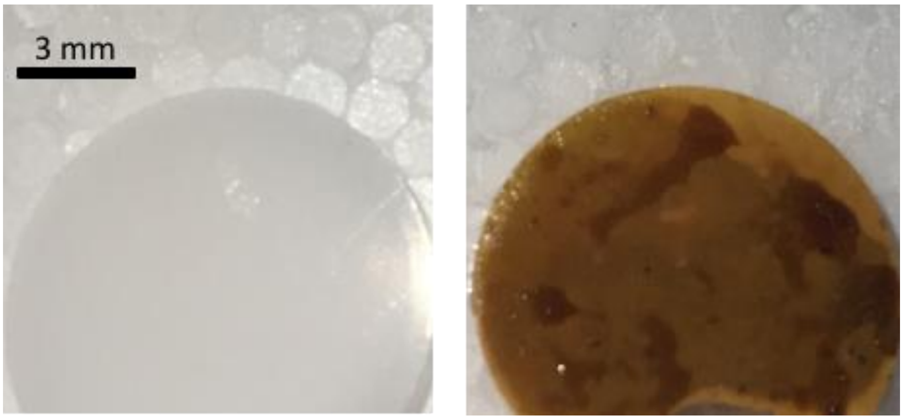

Figure S1. Comparison of the appearance of the MA-precursor solution filled anodized alumina template (**left**) before and (**right**) after microwave heating.

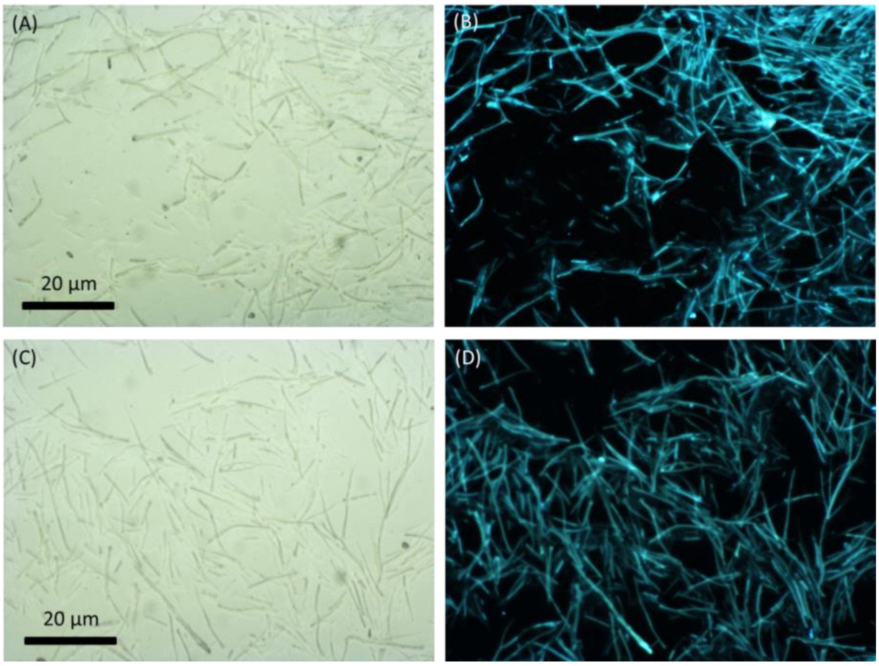

Figure S2. Left Bright field x 100 magnification (scale applies to all) images of MA-CNFs and (**Right**) the corresponding epifluorescence images ($\lambda_{ex.}$ 365 – 420 nm λ_{col} >430 nm). Images of MA-CNFs following 1h bath sonication under conditions of **C** Bright field and **D** $\lambda_{ex.}$365 - 420 nm, λ_{col}>430 nm. Drop cast from aqueous suspension and dried in air.

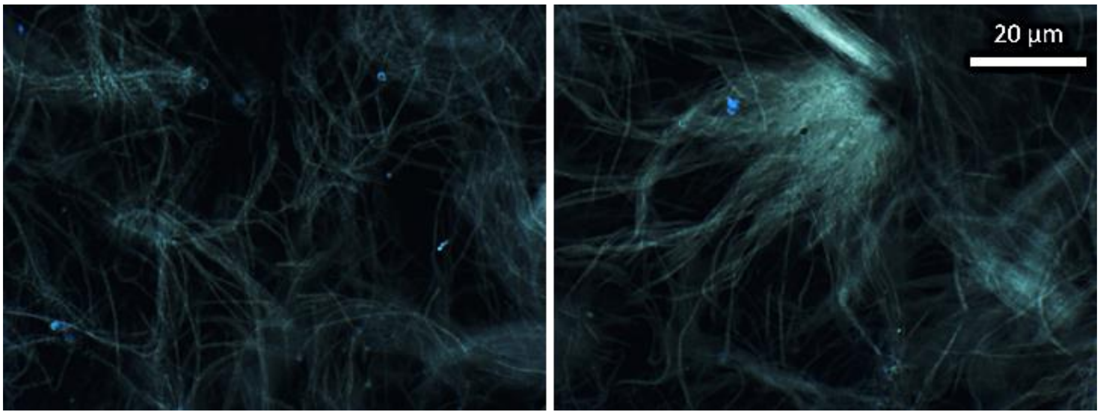

Figure S3. x100 mag. (scale applies to all) epifluorescence images (under $\lambda_{ex.}$ 365 – 420 nm λ_{col} >430 nm) of MA-CNFs prepared by solvothermal methods drop cast from aqueous suspension and dried in air.

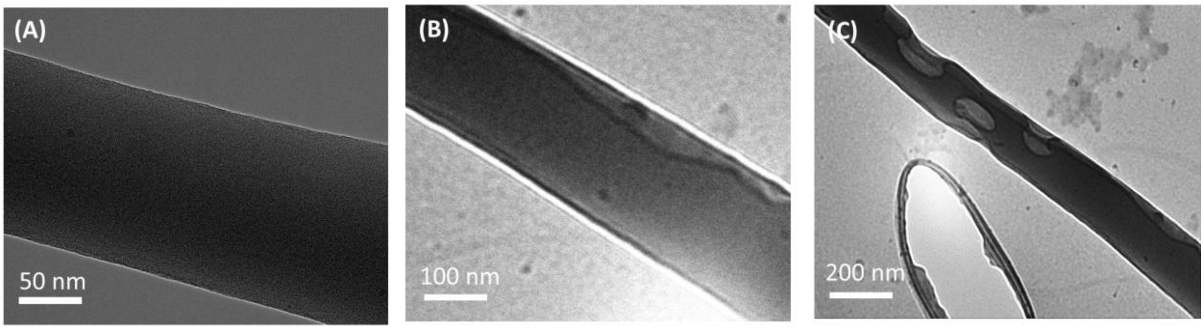

Figure S4. TEM images of MA-CNFs. Drop cast from aqueous suspension and dried in air. **(A)** Deposited on lacey carbon substrate **(B)** and **(C)** Deposited on holey carbon substrate

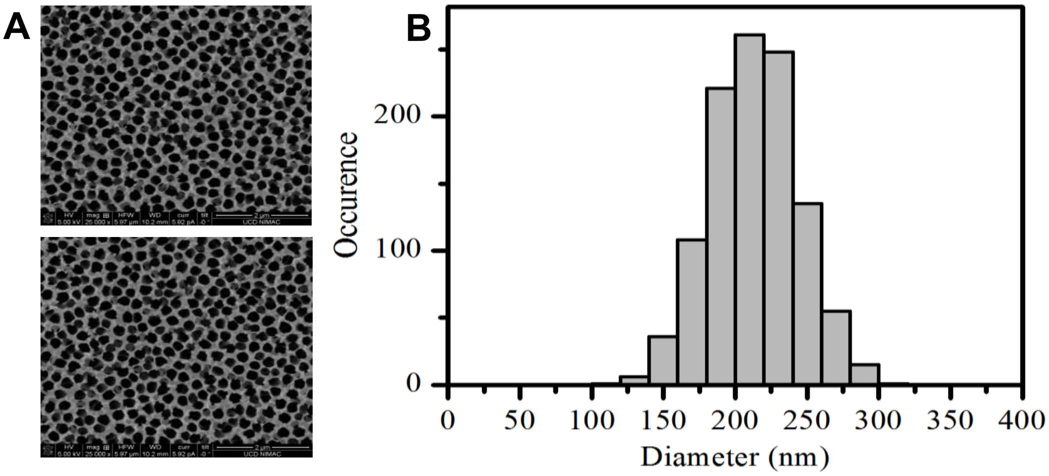

Figure S5. **A** SEM images of empty anodized alumina templates as used in the synthesis of carbon fibers. **B** Size distribution of the diameter of the pore size determined from the SEM images, n = 1087.

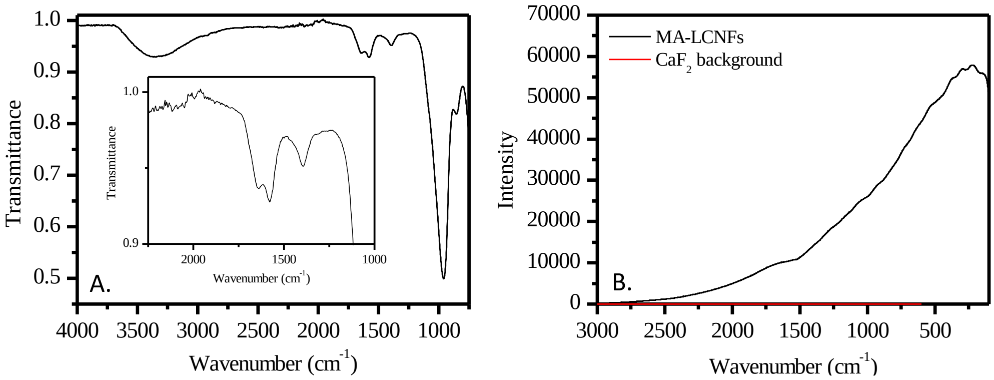

Figure S6. A FTIR spectrum of MA-CNFs deposited on CaF_2 from aqueous suspension. **B** Raman spectrum of MA-CNF, deposited on CaF_2 from aqueous suspension.

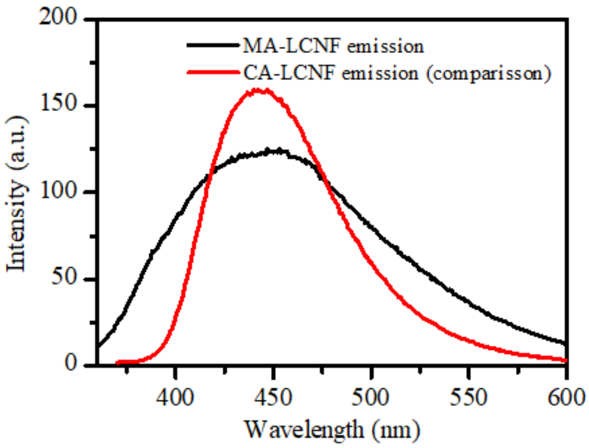

Figure S7 Comparison of the emission spectra of the MA-CNFs **(black)** with the previously reported citric acid based CNFs **(red)**

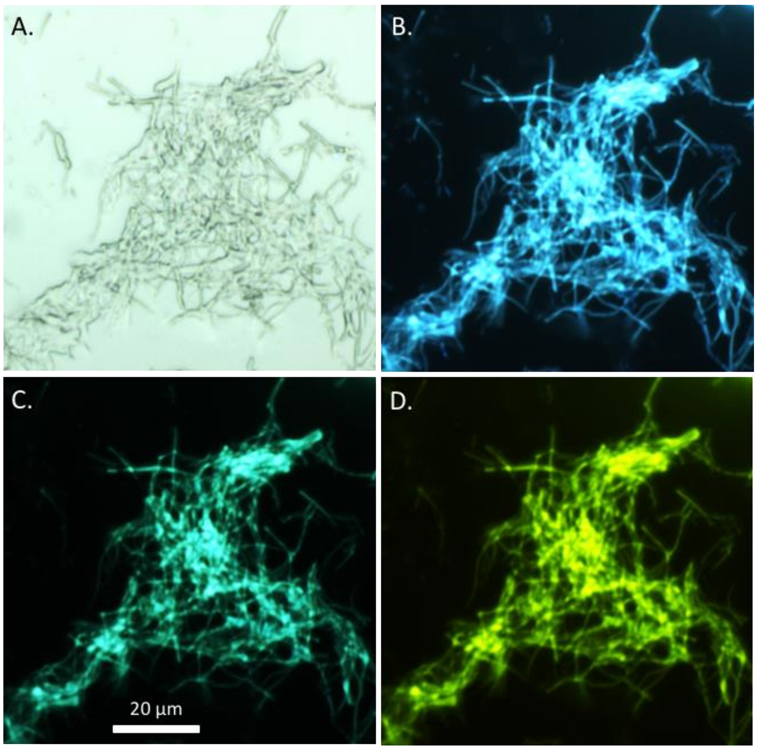

Figure S8. x100 mag. (scale applies to all) images of MA-CNFs drop-cast from aqueous suspension and dried in air under illumination conditions of **A.** Bright field **B.** Epifluorescent images captured under $\lambda_{ex.}$365 - 420 nm, λ_{col}>430 nm **C.** $\lambda_{ex.}$390 - 407 nm, λ_{col}>407 nm **D.** $\lambda_{ex.}$450 - 490 nm, λ_{col}>490 nm.

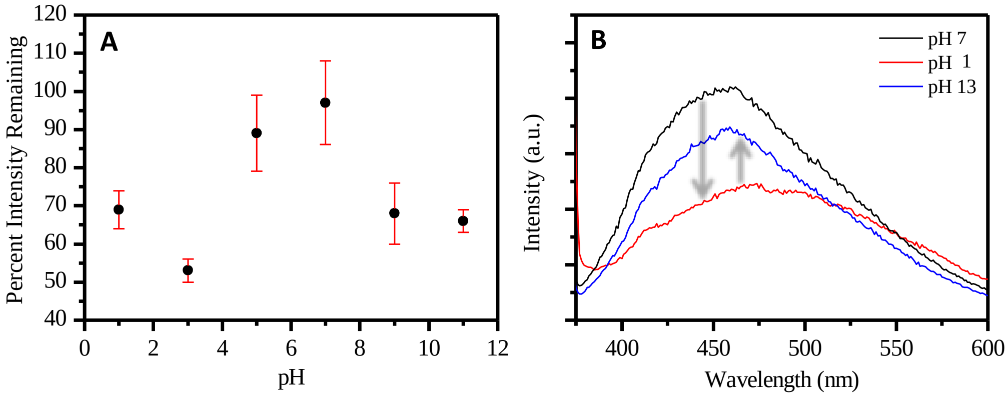

Figure S9. Response of MA-CNFs to changes in their solvent conditions. **A.** pH sensitivity in an aqueous environment. **B.** pH switching in an aqueous environment. Samples had abs of 0.1 at 350 nm.

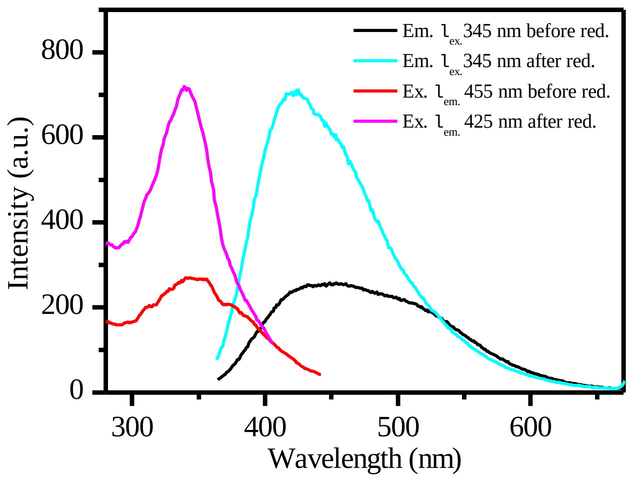

Figure S10. Effect of reduction by sodium borohydride on the emission and excitation of MA-CNF. Samples had abs of 0.1 at 350 nm.

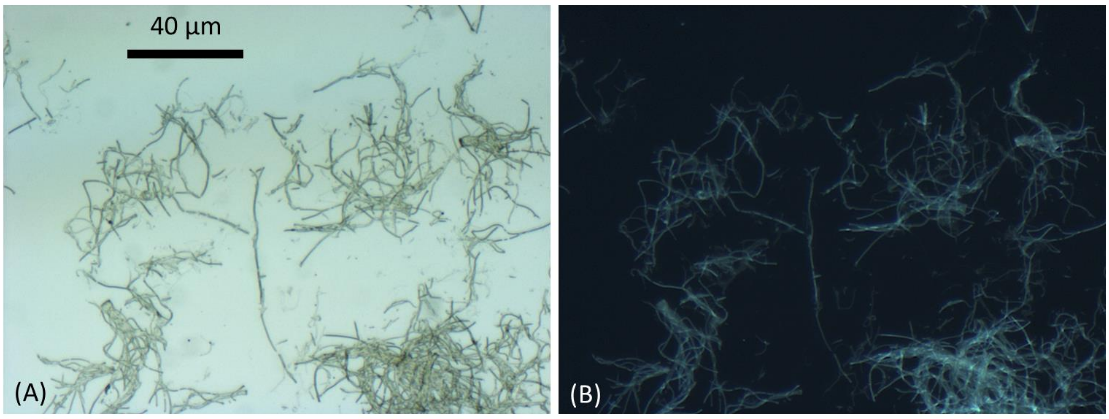

Figure S11. x 100 mag. images of LA-LCNFs under **(A)** Bright field illumination and **(B)** Epifluorescence illumination as shown $\lambda_{ex.}$ 365 – 420 nm λ_{col} >430 nm. Scale applies to all.

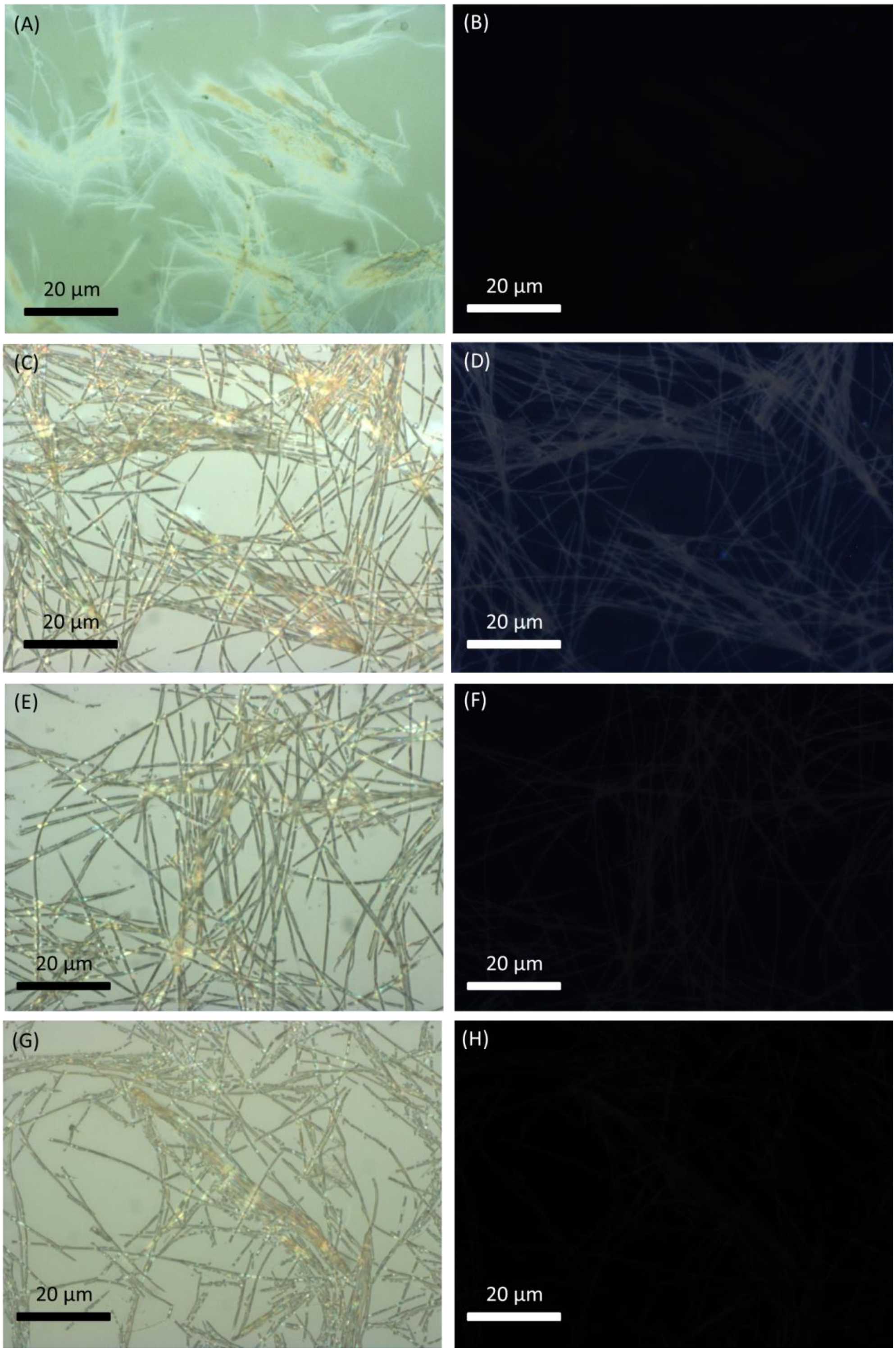

Figure S12. x 100 mag. bright field (**A. C. E.** and **G.**) and epifluorescence images (under $\lambda_{ex.}$ 365 – 420 nm λ_{col} >430 nm) (**B. D. F.** and **H.**) of CNFs prepared without the addition of PEI in the precursor solution **(A)(B)** Lj-CNFs **(C)(D)** Oj-CNFs **(E)(F)** Gj-CNFs and **(G)(H)** CC-CNFs.

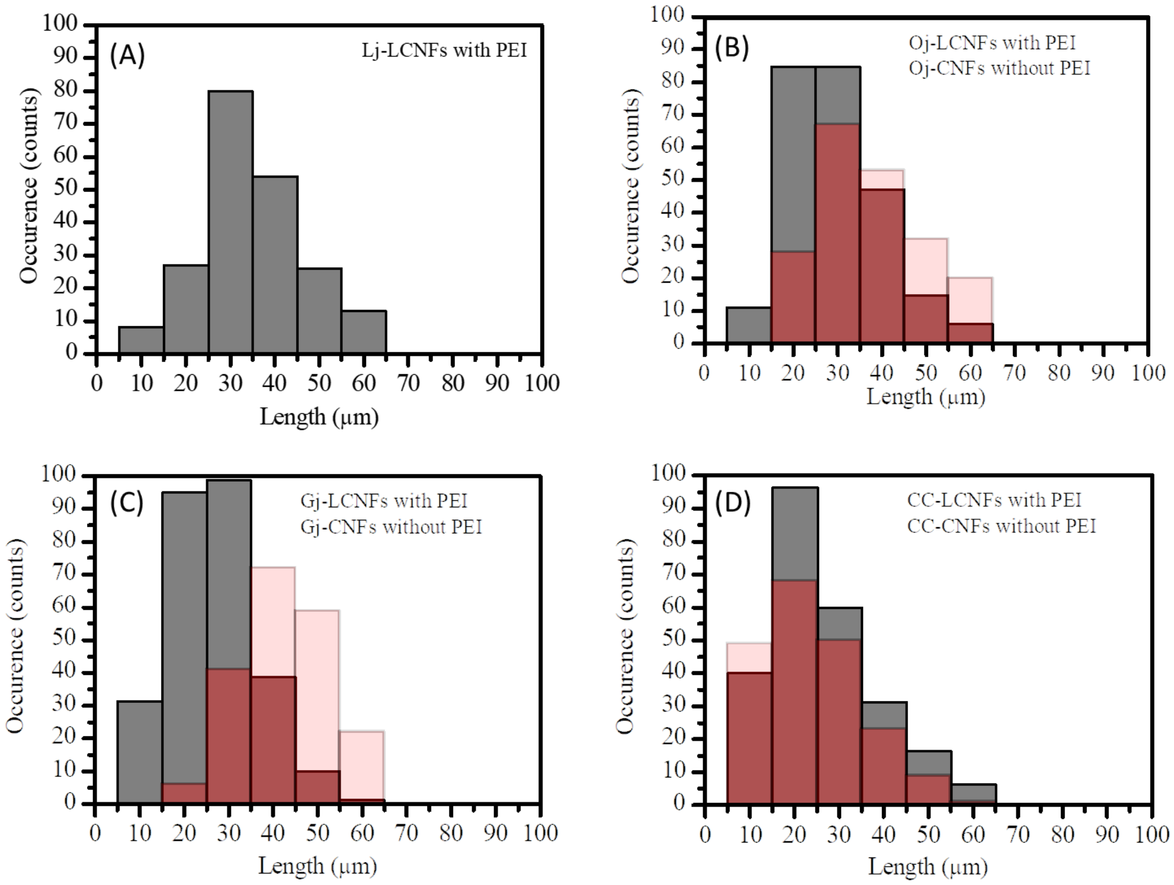

Figure S13. Length distributions of drink-CNFs with and without PEI as measured by bright field images.

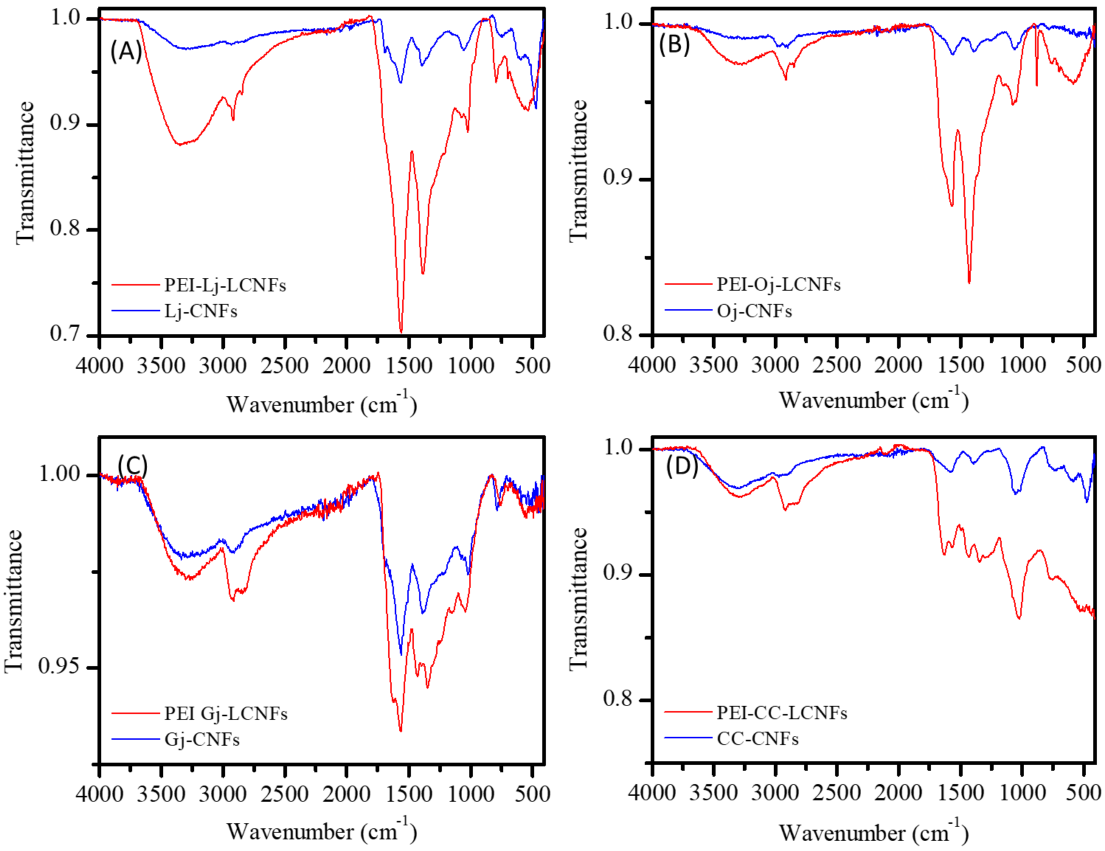

Figure S14. FTIR spectra (solid state) of drink-CNF both with and without the inclusion of PEI in the precursor solution.

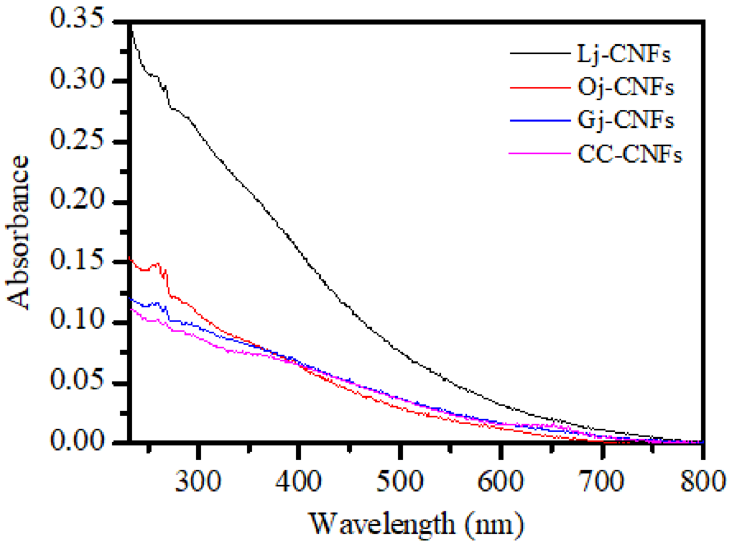

Figure S15. Absorbance spectra of non-PEI containing drink-CNFs in aqueous suspension.

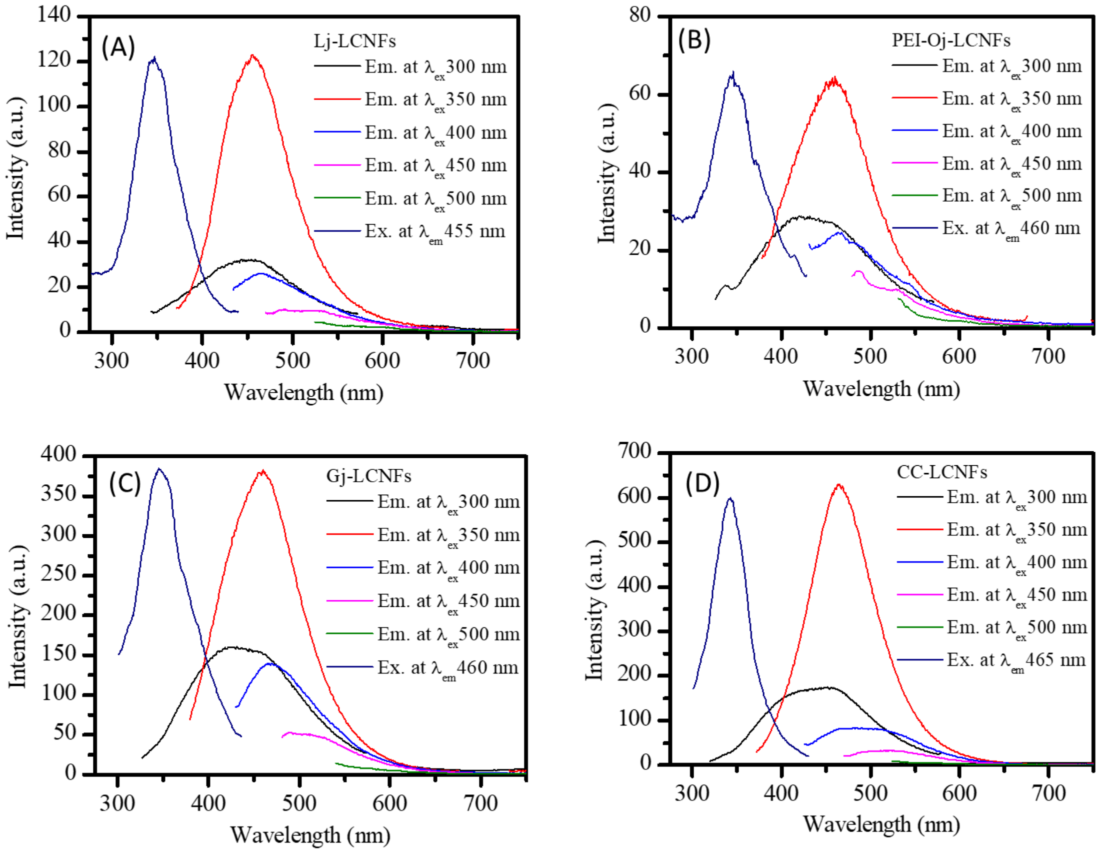

Figure S16. Excitation wavelength dependent emission spectra of all drink-CNFs prepared with PEI in aqueous suspension. Slit width 10 mm 10 mm. PL spectra taken at sample abs 0.1 at 350 nm.

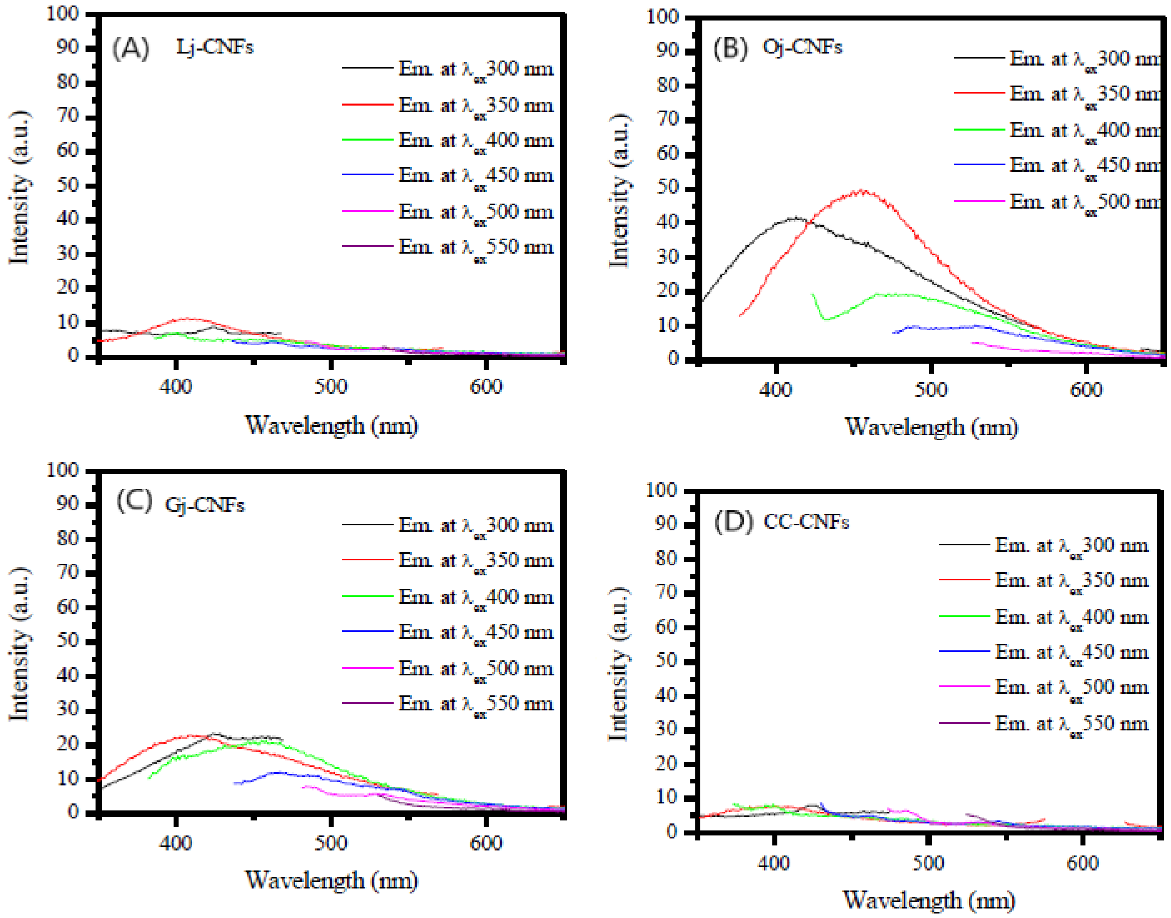

Figure S17. Excitation wavelength dependent emission spectra of all drink-CNFs prepared without PEI in aqueous suspension. Slit width 10 mm 10 mm. PL spectra taken at sample abs 0.1 at 350 nm.

Table S1. Comparison for MA-CNF dimensions with CA-CNF dimensions.

Dimension	Method	CA-CNF	MA-CNF
Length	Epifluorescence imaging	11 ± 5 μm	10 ± 4 μm
Persistence	Epifluorescence imaging	6 ± 3 μm	8 ± 3 μm
Diameter	SEM (pore mouths)	213 ± 31 nm	213 ± 31 nm
Diameter	AFM	207 ± 41 nm	217 ± 32 nm
Diameter	SEM	238 ± 39 nm	
Diameter	TEM		201 ± 27 nm

Table S2. FTIR assignment of MA-CNFs

Peak Position (cm^{-1})	Assignment	Description
3361	O-H/N-H	Alcohol stretch, H-bonded – broadened/ Aliphatic amine stretch (symmetric)
2850	O-H	Acid stretch
1640	C=N	Imine stretch
1670	C=O	Carbonyl stretch
1391	COO-	Symmetric stretch
970	=C-H	Out of plane

Table S3. Reaction mixture composition for preparation of carbon fibers

Sample	*Carbon Precursor*		*PEI*	*H_2O*
	Name	Amount		
MA-CNF	Malic Acid	0.24 g	80 μL	300 μl
LA-CNF	Lactic Acid	0.16	80 μL	300 μl
Lj-CNF	Lemon juice concentrate*	300 μL	80 μL	-
Oj-CNF	Orange juice concentrate*	300 μL	80 μL	-
Gj-CNF	Grapefruit juice concentrate*	300 μL	80 μL	-
CC-CNF	Coca Cola concentrate*	300 μL	80 μL	-

**concentrates were obtained by reducing x10 the volume of as purchased drinks.*

Table S4. Microwave conditions for each of the CNF reactions.

Sample	Time (min)	Temp (°C)	Pressure (bar)	Ramp time (s)	Cool time (s)
MA-CNF	6	250	16	300	110
LA-CNF	6	250	16	300	110
Lj-CNF	6	260	25	360	120
Oj-CNF	6	260	25	360	120
Gj-CNF	6	260	25	360	120
CC-CNF	6	260	25	360	120

Table S5. Elemental analysis of drink-CNFs in atomic percent.

Sample	C (%)	H (%)	N (%)	O (%)	Sample	C (%)	H (%)	N (%)	O (%)*
PEI-Lj-CNFs	48.89	5.90	10.64	34.57	**Lj-CNFs**	53.66	4.48	1.74	40.12
PEI-Oj-CNFs	55.28	6.08	12.63	26.01	**Oj-CNFs**	53.09	4.33	1.68	40.9
PEI-Gj-CNFs	52.22	6.24	14.34	27.2	**Gj-CNFs**	53.82	4.25	1.70	40.23
PEI-CC-CNFs	58.43	6.78	13.56	21.23	**CC-CNFs**	52.51	4.58	0.0	42.91

Table S6. Peak assignments for FTIR of drink-CNFs

With PEI	No PEI				
Peak Position (cm^{-1})	Assignment	Description	**Peak Position (cm^{-1})**	Assignment	Description
3310	O-H/N-H	Alcohol stretch, H-bonded – broadened/ Aliphatic amine stretch (symmetric)	**3310**	O-H	Alcohol stretch
2916	C-H	Alkane stretch (asymmetric)	**2916**	C-H	Alkane stretch (asymmetric)
1633	C=O	Amide stretch	**1633**	C=O	Amide stretch
1569	C=C	Aromatic stretch (1 of 2)	**1569**	C=C	Aromatic stretch
1430	C=C	Aromatic stretch (2 of 2)			
1421	C-H	Alkane bend	**1421**	C-H	Alkane bend
1348	N-H	Aryl stretch			
1236	C-N	Amine stretch			
1145					
1050	C-O	Alcohol stretch	**1050**	C-O	Alcohol stretch
	C-O	Alcohol stretch/ ester stretch			

Table S7. Wavelength positions of emission maxima for drink-CNFs as a function of their wavelength of excitation

CNF	λ_{max} at λ_{ex} 300 nm (nm)	λ_{max} at λ_{ex} 350 nm (nm)	FWHM (nm)	λ_{max} at λ_{ex} 400 nm (nm)	λ_{max} at λ_{ex} 450 nm (nm)	λ_{max} at λ_{ex} 500 nm (nm)
PEI-Lj-CNF	442	455	87	466	534	529
PEI-Oj-CNF	422	460	111	465	530	539
PEI-Gj-CNF	426	460	103	466	514	563
PEI-CC-CNF	457	464	84	500	522	528
